# Tumor lysis syndrome following trastuzumab and pertuzumab for metastatic breast cancer: a case report

**DOI:** 10.1186/s13256-016-0969-5

**Published:** 2016-06-16

**Authors:** C. Baudon, F. P. Duhoux, I. Sinapi, J. L. Canon

**Affiliations:** 1 Oncology-Hematology Services, Grand Hôpital de Charleroi, 6000 Charleroi, Belgium; 2 Medical Oncology, Institut Roi Albert II, Cliniques universitaires Saint-Luc, Université catholique de Louvain, 1200 Brussels, Belgium

**Keywords:** Solid tumor, Trastuzumab, Pertuzumab, Tumor lysis syndrome

## Abstract

**Background:**

Tumor lysis syndrome is a rare and potentially fatal complication of oncologic treatments, especially in solid tumors. To the best of our knowledge, tumor lysis syndrome has never been reported after trastuzumab and pertuzumab combination therapy. Knowledge of risk factors and active prevention proceedings is of utmost importance to avoid fatal outcomes.

**Case presentation:**

We present the case of a chemo-naive 58-year-old Belgian woman developing hypovolemic shock and multiple organ failure due to tumor lysis syndrome after a single dose of trastuzumab and pertuzumab in the context of the treatment of a metastatic breast cancer and resulting in fatal outcome despite optimal management.

**Conclusions:**

Considering that targeted cancer therapies become increasingly effective, oncologists should be extremely cautious when treating patients at high risk of tumor lysis syndrome, even if they are not treated with cytotoxic chemotherapy, and determine appropriate prophylaxis.

## Background

Tumor lysis syndrome (TLS) is a rare complication of oncologic treatments in solid tumors. It results from rapid lysis of tumor cells and release of intracellular materials into the bloodstream. We report a case of TLS which occurred after a single dose of trastuzumab and pertuzumab in the context of metastatic breast cancer in a chemo-naive patient. To the best of our knowledge, TLS has never been reported after trastuzumab and pertuzumab combination therapy.

## Case presentation

A 58-year-old Belgian woman was admitted to our hospital with complaints of anorexia, fatigue, and dyspnea. A right breast cancer had been diagnosed 1 month before. It was an invasive grade III ductal carcinoma, with no expression of hormone receptors and with human epidermal growth factor 2 (HER2) amplification. The disease was locally advanced with massive invasion of the chest wall, bilateral axillary lymph node involvement, pulmonary metastases, and bulky liver metastases. A weekly paclitaxel, trastuzumab, and pertuzumab schedule was planned as first-line chemotherapy. Our patient had a medical history of pulmonary tuberculosis.

At admission, pulmonary examination showed hypoventilation of the right pulmonary basis. A right pleural effusion was demonstrated by a computed tomography (CT) scan, complicated by a right inferior pneumonia. The chest wound was infected by multiple Gram-negative bacilli. Pleural effusion was punctured, and its culture was sterile. Blood tests at admission showed a high C-reactive protein level (126 mg/L), hyperuricemia (55 mg/dL), high lactate dehydrogenase (983 U/L) and uric acid (12 mg/dL) levels and decreased glomerular filtration rate (53 mL/min), probably due to dehydration. Her urine analysis was normal. Our patient received empiric antibiotic therapy with amoxicillin and clavulanic acid.

Four days later she developed respiratory distress. No pulmonary embolism was demonstrated on the CT scan performed in the emergency department, and symptoms were attributed to the pleural effusion and right pneumonia. Our patient was treated 1 day later with a first course of trastuzumab and pertuzumab. The infusion of paclitaxel was delayed due to the infectious context and was planned to be given later.

On day 2 of the first course of trastuzumab and pertuzumab, she developed refractory hypovolemic shock. Her blood tests showed grade 2 hyperkaliemia (6.4 mmol/L), hyperuricemia (11.4 mg/dL), high lactate dehydrogenase (2473 U/L), grade 3 hypocalcemia (1.73 mmol/L), hyperphosphatemia (1.64 mmol/L), and worsening of the acute renal failure (38 mL/min glomerular filtration rate). Blood gas analysis revealed metabolic acidosis with a pH of 7.11 and lactate at 10.9 mmol/L. A diagnosis of TLS induced by the targeted therapy was made. She was admitted in the intensive care unit where she received vigorous volume expansion and vasopressors. Despite optimal management, her laboratory values and hypovolemic shock did not improve and she died 2 days later due to multiple organ failure.

## Discussion

TLS is a rare entity. Its incidence is not known but may be less than 0.3 % in solid tumors in a single-center study [1]. It is a potentially fatal oncologic emergency characterized by severe electrolyte and metabolite abnormalities secondary to rapid and massive lysis of tumor cells and release of intracellular components into the bloodstream, overwhelming the excretion capacities of the kidneys and leading to hyperuricemia, hyperkalemia, hyperphosphatemia, and secondary hypocalcaemia. These electrolytic abnormalities explain the clinical symptoms. Hyperuricemia and hyperphosphatemia can lead to acute renal injury by way of uric acid precipitation and calcium phosphate deposition in the renal tubules. Hypocalcemia and hyperkalemia potentially lead to electrocardiogram (ECG) abnormalities and cardiac arrhythmias, neuromuscular symptoms, and seizures.

Our patient responded to the diagnostic criteria of laboratory TLS (detailed in Table 1), as the three laboratory criteria were present and the worsening of acute renal failure to grade I responds to the definition of clinical TLS criteria established by Cairo and Bishop in 2004 [2] (detailed in Table 2). It is important to note that TLS is defined as the presence of at least two or more biochemical variables within the 3 days before chemotherapy or 7 days after chemotherapy in the face of adequate hydration and use of uric acid-lowering agent. Of note, neither targeted treatments nor local therapies are excluded from this definition. Symptoms are observed more commonly within the 12 to 72 hours after the initiation of the treatment [2].

**Table 1:** Laboratory Tumor Lysis Syndrome criteria

Variable	Value	Change from baseline value
Uric acid	≥8 mg/dL (476 mmol/L)	25 % increase
Potassium	≥6 mEq/L (or 6 mmol/L)	25 % increase
Phosphorus	≥4.5 mg/dL (1.45 mmol/L)	25 % increase
Calcium	≤7 md/dl (1.75 mmol/L)	25 % increase

**Table 2:** 2004 Cairo and Bishop criteria for Tumor Lysis Syndrome

Variable	Grade 0	Grade I	Grade II	Grade III	Grade IV	Grade V
Creatinine	None	1.5 X ULN	˃1.5–3.0 X ULN	˃3.0–6.0 X ULN	˃6.0 X ULN	Death
Cardiac arrhythmia	None	Intervention not indicated	Nonurgent medical intervention indicated	Symptomatic and incompletely controlled medically or controlled with device (e.g., defibrillator)	Life-threatening (e.g., arrhythmia associated with CHF, hypotension, syncope, shock)	Death
Seizures	None		One brief, generalized seizure; seizure(s) well controlled by anticonvulsants, or infrequent focal motor seizures not interfering with ADL	Seizures in which consciousness is altered; poorly controlled seizure disorder; with breakthrough generalized seizures despite medical intervention	Seizures of any kind which are prolonged, repetitive, or difficult to control (e.g., status epilepticus, intractable epilepsy)	Death

*ADL* activities of daily living, *CHF* congestive heart failure, *ULN* upper level of normal

Moreover, patients at risk of TLS are classified in low-, intermediate- and high-risk categories [3].

Solid tumors are by default classified in the low-risk category but the presence of pre-existing risk factors, high tumor burden, high sensitivity to antitumoral treatment, and efficacy of the therapies could classify them in the intermediate- or high-risk group [3, 4].

Tumor and patient-related risk factors of TLS in solid tumors previously described are bulky disease, extensive metastases, high proliferative rate, high sensitivity to anticancer therapy, type of anticancer therapy (e.g., using a combination therapy), elevated LDH, serum creatinine, uric acid, phosphate at baseline, low urinary flow, dehydration, preexisting nephropathy, extrinsic compression of the urinary tract by the tumor, and exposure to nephrotoxins. Some authors think that metastatic disease and particularly metastases in the liver should be a relevant risk factor, irrespective of the liver function, and maybe due to impaired uric acid metabolism and high purine pools [5, 6].

Our patient presented multiple risk factors: elevated uric acid, elevated LDH, elevated creatinine, exposure to nephrotoxins (contrast agents 2 days before), extensive liver metastases, and bulky disease.

Assessment of risk factors is of utmost importance because recognition of high risk of TLS should lead to more active prevention. Regarding this patient, TLS is not usual in solid tumors, and in particular in breast tumors but the high effectiveness of the trastuzumab and pertuzumab association could have been the trigger. A fatal outcome might have been avoided in this case, especially as it has been demonstrated that clinical TLS (but not laboratory TLS) is associated with an increased death rate (83 % vs 24 %; *p* < 0.001) [7]. Moreover, development of acute kidney injury associated with TLS is a strong predictor of death [8]. Irrespective of the cancer type, there is a 20–50 % increase in mortality for undiagnosed or late-diagnosed TLS in solid tumors [9]. The best management for TLS is prevention.

TLS prophylaxis should depend on the risk group in which a patient is classified. Low-risk disease should simply be monitored and no prophylaxis is required. While the prophylactic approach for intermediate-risk patients is still not clearly defined [6], it is recommended to monitor these cases for TLS; they should receive increased hydration (3 l/m^2^ per day) and be administered allopurinol (100–300 mg, orally, every 8 hours, daily). In the group at high risk of developing TLS, patients should be monitored more frequently (possibly in an intensive care unit), receive increased hydration (3 l/m^2^ per day) unless evidence of renal insufficiency and oliguria, loop diuretics if necessary, and rasburicase (0.1–0.2 mg/kg for one dose and repeated if needed seems to be the most cost-effective approach) [3]. Urine alkalinization is no longer recommended: it has not been shown to be superior to the administration of normal saline alone and might even be harmful [4, 10].

Unfortunately, despite appropriate prophylactic measures, about 3 to 5 % of high-risk patients receiving chemotherapy will develop TLS [11].

TLS was previously well described in hematologic malignancies, but as treatments become more efficient, it is becoming increasingly frequent in solid tumors, which previously had been rarely associated with this complication [12]. Chemotherapy is the most frequent etiology of TLS (58 %), but TLS was also described with multiple anticancer treatments such as radiotherapy, restrictive surgical procedures, immunotherapy, endocrine therapy, radiofrequency ablation, chemoembolization, bisphophonates, and glucocorticoid therapy alone. In addition, TLS can occur spontaneously due to tumor necrosis prior to any therapy [5]. Case reports involving targeted therapies such as tyrosine kinase inhibitors or cyclin-dependent kinases, where TLS was a dose-limiting toxicity in phase I trials, and monoclonal antibodies are increasingly often described [13]. One case of TLS has been reported after trastuzumab alone [14]. To the best of our knowledge, TLS has never been described with the association of trastuzumab and pertuzumab. As shown in the NeoSphere trial, this association is highly effective, as without any chemotherapy, its use in operable, locally advanced or inflammatory breast cancer leads to a pathological complete response rate (pCR) of 16.8 % in the breast (Gianni *et al*.) [15]. Based on these encouraging results, a randomized phase II study led by the EORTC Cancer in the Elderly Task Force and the EORTC Breast Cancer Group is currently investigating the use of trastuzumab and pertuzumab (with or without metronomic chemotherapy) in elderly metastatic breast cancer patients. The use of targeted therapies alone in breast cancer is thus becoming increasingly popular because of its relative tolerance and high efficacy. As exemplified in this case report, oncologists should be cautious when using these drugs in patients at high risk of TLS.

## Conclusions

We here describe the first case of TLS in a patient with metastatic breast cancer with the association of trastuzumab and pertuzumab. As targeted cancer therapies become increasingly effective, oncologists treating breast cancer patients should be extremely cautious when treating patients at high risk of TLS, even if they are not treated with cytotoxic chemotherapy.

As TLS can unfortunately lead to a fatal outcome, physicians should take into account the risk of TLS before the initiation of any anticancer treatment, and determine appropriate prophylaxis.
